# Variations in the Spatial Distribution of the Amplitude of Surface Electromyograms Are Unlikely Explained by Changes in the Length of Medial Gastrocnemius Fibres with Knee Joint Angle

**DOI:** 10.1371/journal.pone.0126888

**Published:** 2015-05-22

**Authors:** Carolina Avancini, Liliam F. de Oliveira, Luciano L. Menegaldo, Taian M. Vieira

**Affiliations:** 1 Programa de Engenharia Biomédica (COPPE), Universidade Federal do Rio de Janeiro, Rio de Janeiro, RJ, Brasil; 2 Escola de Educação Física e Desportos, Universidade Federal do Rio de Janeiro, Rio de Janeiro, RJ, Brasil; 3 Laboratorio di Ingegneria del Sistema Neuromuscolare (LISiN), Politecnico di Torino, Torino, TO, Italia; Northwestern University Feinberg School of Medicine, UNITED STATES

## Abstract

This study investigates whether knee position affects the amplitude distribution of surface electromyogram (EMG) in the medial gastrocnemius (MG) muscle. Of further concern is understanding whether knee-induced changes in EMG amplitude distribution are associated with regional changes in MG fibre length. Fifteen surface EMGs were acquired proximo-distally from the MG muscle while 22 (13 male) healthy participants (age range: 23–47 years) exerted isometric plantar flexion at 60% of their maximal effort, with knee fully extended and at 90 degrees flexion. The number of channels providing EMGs with greatest amplitude, their relative proximo-distal position and the EMG amplitude averaged over channels were considered to characterise changes in myoelectric activity with knee position. From ultrasound images, collected at rest, fibre length, pennation angle and fat thickness were computed for MG proximo-distal regions. Surface EMGs detected with knee flexed were on average five times smaller than those collected during knee extended. However, during knee flexed, relatively larger EMGs were detected by a dramatically greater number of channels, centred at the MG more proximal regions. Variation in knee position at rest did not affect the proximo-distal values obtained for MG fibre length, pennation angle and fat thickness. Our main findings revealed that, with knee flexion: i) there is a redistribution of activity within the whole MG muscle; ii) EMGs detected locally unlikely suffice to characterise the changes in the neural drive to MG during isometric contractions at knee fully extended and 90 degrees flexed positions; iii) sources other than fibre length may substantially contribute to determining the net, MG activation.

## Introduction

The triceps surae muscle, composed by the two gastrocnemius heads and the soleus muscle, is the chief ankle plantar flexor. Approximately 70% of the plantar flexion torque applied at the ankle results exclusively from the triceps surae activation [1]. However, due to their anatomical differences, gastrocnemius and soleus muscles provide different relative contributions to the ankle plantar flexion torque. Differently from soleus, the gastrocnemius muscles span both the ankle and knee joints; their force vectors contribute to both ankle extension and knee flexion torque. As a consequence, the relative contribution of each head of the triceps surae to plantar flexion torque changes with the knee joint position.

Mechanically, the gastrocnemius muscles may produce substantially greater plantar flexion torque when the knee is at progressively more extended positions. When the knee is fully extended, previous estimates suggest the plantar flexion torque produced by the gastrocnemius muscle amounts to ~45% of the total, plantar flexion torque [1]. This figure decreases to ~30% for knee flexed positions [2]. The smaller values of plantar flexion torque observed for the more flexed knee positions are typically attributed to the gastrocnemius force-length curve [3]. Specifically, for knee joint angles smaller than that corresponding to full extension, the gastrocnemius fibres are on average shorter than their optimal length for force production [4]. Presuming the neural drive to gastrocnemius motor neurons remains constant for different knee joint positions, the muscle mechanical output is therefore expected to decrease with knee flexion.

Through the recording of surface electromyograms (EMG), previous studies have consistently reported a differentiated degree of activation of the gastrocnemius muscle for different, knee joint positions [5, 6, 7]. These differences in activation seem to manifest equally during both dynamic and isometric contractions. Tamaki and co-workers, for example, recorded surface EMGs from the gastrocnemius muscle while subjects moved their ankle into plantar flexion, at three different speeds and at three knee joint angles [8]. Regardless of the contraction speed, these authors observed significantly smaller peak values of integrated EMGs for the more flexed knee positions. Smaller values of EMG amplitude have been similarly documented for the gastrocnemius muscle during isometric plantar flexion contractions performed with knee flexed rather than extended [5]. Such decrease in EMG amplitude with knee flexion has been conceived as a strategy of the nervous system to more efficiently distribute the neural drive between plantar flexors [9]. In virtue of the suboptimal length of gastrocnemius fibres at knee-flexed positions, the relative active contribution of this muscle to the production of plantar flexion torque likely decreases with knee flexion.

Previous studies reporting the effect of fibre length on the gastrocnemius mechanical efficiency and activation have conceived the muscle as a homogeneous medium [10, 11]. On the other hand, anatomical and electrophysiological evidence suggests the changes in architecture and activation may distribute unevenly within the gastrocnemius muscle. For example, spatial changes in fibre length within the medial gastrocnemius (MG) muscle were observed during walking and running [12] and with multi-joint leg extension [3]. Similarly, imaging techniques and electromyography have consistently revealed a significant differential pattern of activation between proximal and distal gastrocnemius regions. These regional variations in activation have been reported following dynamic plantar flexion contractions at different intensities [13], during quiet standing [14], during electrically elicited contractions [15], with changes in ankle force direction [16] and with fatigue [17, 18]. Whether the nervous system accounts for anatomical inhomogeneities within the gastrocnemius muscle to shape activation with the changes in knee position remains however an open issue. If fibre length is the key parameter shaping activation, then, the gastrocnemius regions showing smallest reductions in fibre length with knee flexion may be activated most strongly.

In this study we therefore use ultrasound to address the question: does the amplitude of surface EMGs detected from different MG regions change with knee position? If it does, then we further investigate whether the changes in EMG amplitude distribution are associated with changes in fibre length within the MG muscle. If the nervous system redistributes the neural drive to the MG muscle predominantly according to the length of its fibres, in agreement with previous accounts on changes in EMG amplitude with knee position [9, 7], we expect to observe greatest reductions in EMG amplitude where reductions in fibre length are greatest.

## Materials and Methods

### Subjects

Twenty-two healthy (13 male) volunteers participated in the study (range values; age: 23–47 years; height: 150–195 cm; body mass: 44–90 Kg). Participants were instructed about the experimental procedures and provided written, informed consent prior to participation. Experimental procedures conformed to the standards set by the latest revision of the Declaration of Helsinki and were approved by the institutional ethics committee (HUCFF/UFRJ—127/2013).

### Quantifying gastrocnemius architecture

Before acquisition of EMGs and of torque data, gastrocnemius architecture was carefully analysed to investigate whether anatomical factors affected the degree and the distribution of MG activity. Of particular interest was the effect of pennation angle [19], of fibre length [20] and of fat thickness [21] on the amplitude distribution of surface EMGs. These variables were therefore estimated for knee flexed and extended positions from ultrasound images (10 MHz B mode linear probe with 40 mm width, 70% gain and 7 cm depth view; MYLab25 Gold; ESAOTE S.p.A., Italy). All images were taken by an expert therapist while participants were at rest, lying on a padded bed in prone position. The ankle and knee joint angles were controlled with the assistance of a goniometer. The specific experimental procedures considered to estimate MG architecture are detailed below.

Anatomical MG sites were first identified with ultrasound imaging and marked on the skin. Initially, the insertion of the Achilles tendon to the calcaneous bone was identified with the ultrasound probe oriented longitudinally to the leg. After that, with the probe at the same orientation, the medial femoral condyle was identified. The distance between the medial femoral condyle and Achilles tendon insertion was considered to quantify the muscle-tendon length. At 30% of the muscle-tendon length, the lateral and medial boundaries of MG were identified with the probe oriented transversally to the leg. A line was then drawn from the half way point between MG borders to a line drawn between the femoral condyles. With the probe oriented along this line, the MG-Achilles tendon junction was located and marked on skin [22]. Finally, the region defined from the femoral condyle to the myotendinous junction was considered for the acquisition of panoramic image from the MG muscle. Two panoramic images were collected for each of the two knee positions, flexed and extended.

**Fig 1:**
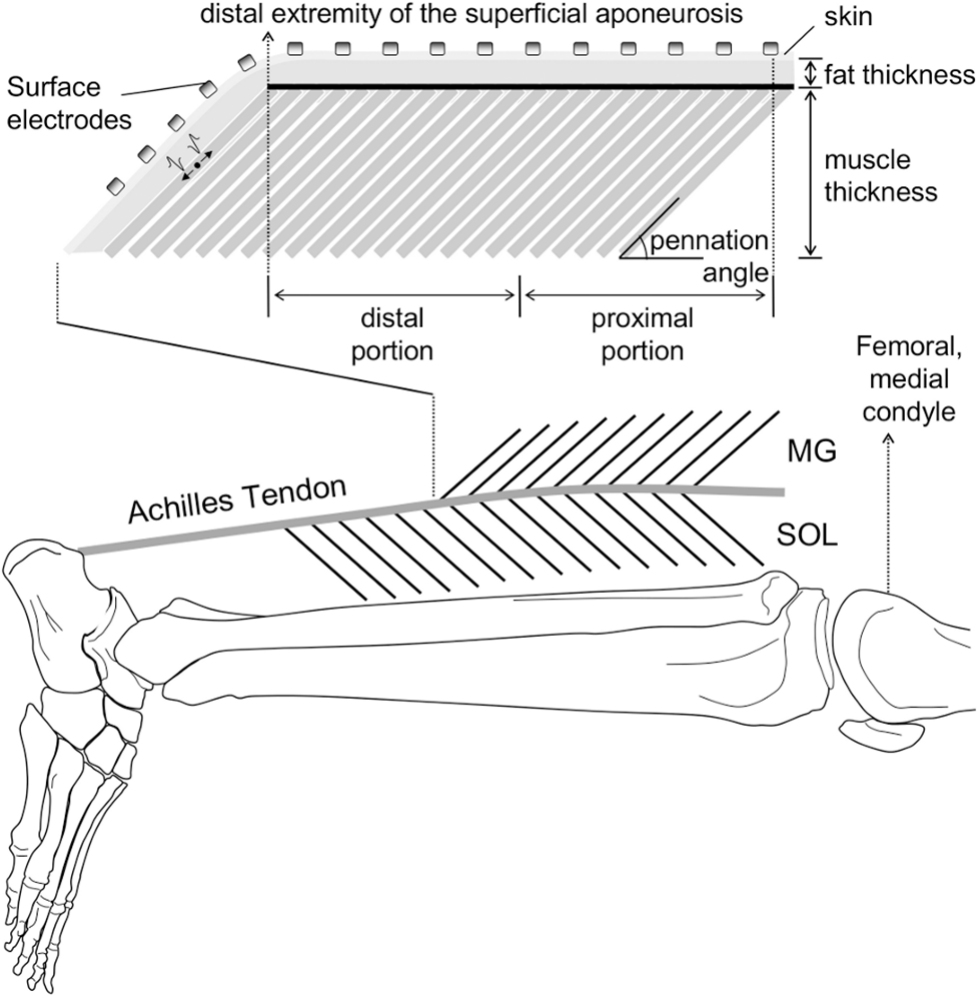
Electrodes positioning and gastrocnemius architecture. A schematic illustration of the relative position of surface electrodes on the medial gastrocnemius (MG) muscle is shown. The parameters considered to characterise architectural differences between the MG proximal and distal regions are further illustrated in the figure; pennation angle, fibre length and fat thickness. Proximal and distal MG regions were respectively defined as the proximal and distal half of the distance between the distal extremity of the superficial aponeurosis and the most proximal electrode. Only the surface EMGs detected by electrodes positioned in correspondence of the superficial aponeurosis were retained for analysis.

Key architectural MG parameters were quantified from the panoramic images, acquired along the entire MG muscle length. These images were analysed with the *Image J* software (National Institute of Health, version 1.42, Bethesda, Maryland, USA). First, the length of the MG region sampled by the surface electrodes was quantified as the distance between the skin region located over the distal extremity of the MG superficial aponeurosis and the most proximal electrode (see next subsection). Then, based on this length, the MG muscle was divided into two portions; proximal and distal portions (Fig 1). MG pennation angle and fat thickness were quantified at the first and second thirds of each portion and then averaged, resulting into a single value for each muscle portion. The pennation angle was estimated as the angle between MG fascicles and the deep aponeurosis. The thickness of the subcutaneous fat tissue was quantified as the distance between the skin/fat and the fat/superficial aponeurosis interfaces [20]. Fibre length was estimated as the average length of lines drawn along clearly visible fascicles located nearby two midpoints of ultrasound images, both extending from superficial to deep aponeurosis.

### Experimental protocol

After collection of ultrasound images, isometric plantar flexions were applied with participants sitting on a dynamometer chair (Biodex System 4, New York, USA). The axis of rotation of the dynamometer was aligned as coaxially as possible with the axis of rotation of the right ankle, defined as the line connecting the tips of medial and lateral malleolus [23]. This alignment was approximated with the assistance of a laser pen, pointing from the centre of the dynamometer’s axis of rotation to the most prominent region of the lateral malleolus. After aligning and securing the right foot to the dynamometer footplate, volunteers were instructed to exert two maximal isometric voluntary contractions (MVCs) for the knee fully extended and two for the knee flexed at 90 deg, lasting 5 s each. During contractions the ankle joint was held in neutral (90 deg) position. The highest peak torque value was retained as representative of the individuals’ maximal effort in each knee position. A rest period of at least 1 min was provided between MVCs. Verbal encouragement assisted participants in reaching their highest plantar flexion torque. At least 2 minutes after the maximal attempts, participants were asked to exert one isometric plantar flexion at 60% MVC with the knee fully extended and one contraction with the knee flexed to 90 deg. Contractions lasted 10 s each, with a rest period of 1 min in-between. Visual feedback of ankle torque was provided to ensure participants kept their plantar flexion effort within 10% of the target level.

### Electrode placement and EMG recordings

Surface EMGs were detected from multiple skin regions covering the MG muscle with a flexible, adhesive array of electrodes. Such array (16 silver-bar electrodes; 10x1 mm; 10 mm inter-electrode distance; Spes Medica, Battipaglia, Italy) was positioned parallel to the MG longitudinal axis (Fig 1). The most proximal electrode was positioned as proximally as possible to the femoral condyle, to avoid folding the array when subjects flexed their knees. Conductive paste (TEN 20 Conductive Paste, Weaver) ensured the electrical contact between electrodes and skin. The reference electrode was placed on the lateral malleolus of the contralateral limb. Before positioning electrodes, the skin was carefully shaved and cleaned with abrasive paste to reduce skin impedance.

Surface EMGs were recorded in single-differential derivation, with 16 electrodes providing a total of 15 single-differential EMGs. To ensure the highest signal to noise ratio without saturation, all signals were amplified by a variable factor, ranging from 2,000 to 5,000 (multi-channel amplifier; 10–900 Hz anti-aliasing filter; CMRR>100 dB; EMG-USB2, OTBioeletronica, Turin, Italy). EMGs were digitalised at 2048 Samples/s with a 12 bits A/D converter. The torque signal provided by the dynamometer machine was sampled synchronously with the EMGs. All signals were inspected prior to acquisition to check and correct for contact problems and power line interference.

### Assessing the spatial distribution of EMG amplitude

The distribution of the amplitude of surface EMGs collected from the MG muscle was quantified for each subject and knee position. First, all EMGs were filtered with a second order, band-pass filter (Butterworth, 15–350 Hz cut-off frequencies). After that, the root mean square (RMS) value was computed over the whole record duration (10 s), separately for each of the 15 channels (i.e., each pair of electrodes). Only channels located on skin regions covering the superficial aponeurosis (Fig 1) and detecting surface EMGs with RMS amplitude greater than 70% of the maximum amplitude [24] were retained for analysis; these channels were termed *active channels*. Finally, from the RMS values obtained for these channels, three indexes were computed: i) the global EMG amplitude, defined as the RMS value averaged over the *active channels*; ii) the barycentre coordinate of the *active channels*, which indicate the mean position of the RMS distribution along the muscle proximo-distal axis and; iii) the number of *active channels*, which denote the spread of the RMS amplitude distribution on the skin.

**Fig 2:**
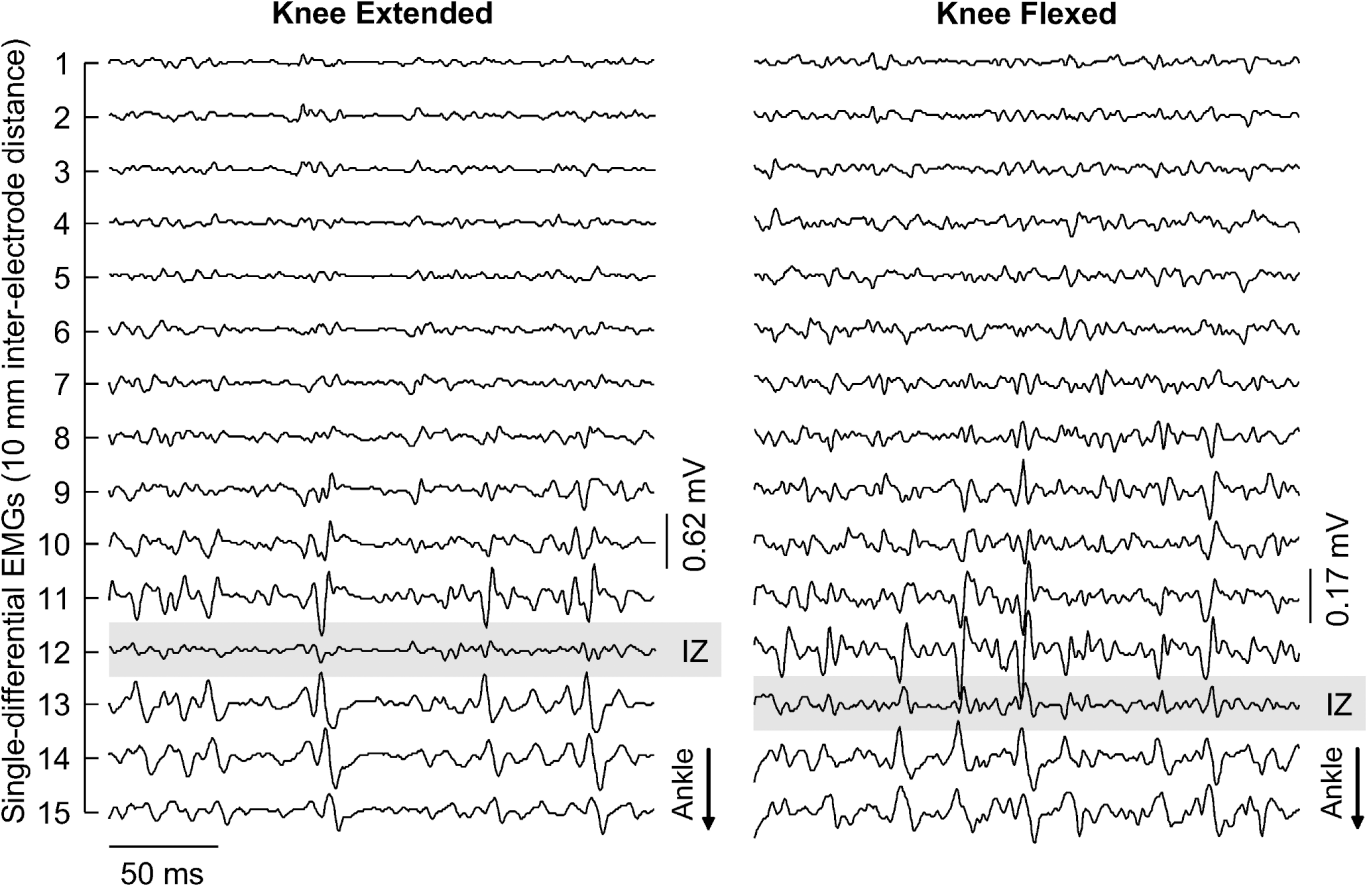
Displacement of innervation zone with knee flexion. Short epochs (250 ms) of the 15 single-differential EMGs collected from a single participant are shown. Signals in the left and right panels were obtained during knee extended and knee flexed positions, respectively. Propagating potentials are observed in the most distal channels, which were covering the most distal MG fibres. The channel in the array positioned most closely to the innervation zone of the muscle distal fibres is indicated with grey, shaded rectangles. Note the innervation zone moved distally from knee extended to knee flexed position.

Specific procedures were applied to normalise each of the three indexes considered. The global EMG amplitude was normalised with respect to the maximal RMS amplitude across all channels, obtained at 100% MVC during the knee extended condition. The barycentre coordinate was calculated from the most proximal electrode in the grid and represented as a percentage of the distance between the femoral condyle and the distal extremity of the superficial aponeurosis, measured with the knee extended. The number of *active channels* was ultimately normalised with respect to the number of channels located over the superficial aponeurosis. With knee flexion, the position of channels in relation to the superficial aponeurosis may change. For this reason, changes in the number of channels located in the skin region over the superficial aponeurosis were assessed through changes in the innervation zone position. Innervation zone was identified through visual inspection of surface EMGs [21]. Whenever a distal shift in the innervation zone position with knee flexion was observed (Fig 2), the number of channels considered for normalisation of the *active channels* was increased; one channel per centimetre shift.

### Statistical analysis

After ensuring the homogeneity of variance with Levene’s test (W values greater than 0.2 for all architecture variables considered) and the data Gaussian distribution (Shapiro-Wilk statistics p>0.075 for all cases), parametric tests were considered to assess the changes in MG architecture with variation in knee joint angle. Two-way analysis of variance (ANOVA) was used to test for the differences in fibre length, pennation angle and fat thickness of MG muscle between and within knee positions and muscle portions. Gaussianity and homogeneity of variance were however not confirmed for the MVC torque scores and for the electromyographic variables. Wilcoxon rank sum test was applied to compare the MVC torque value and the global RMS value, the barycenter longitudinal position and the number of *active channels* obtained for knee extended and flexed positions. All analyses were carried out with IBM SPSS Statistics 20.0 (IBM SPSS, Chicago, USA) and the level of significance was set at *P* < 0.05.

## Results

The potential to produce maximal scores of plantar flexion torque depended on the knee position. Average plantar flexion torque at 100% MVC was significantly greater with knee extended (131± 51 Nm) than with knee flexed (104 ± 53 Nm; Wilcoxon test; *P* = 0.009; *N* = 22 subjects). As outlined below, in addition to the MVC scores, knee flexion affected the amplitude distribution of surface EMGs during sub-maximal contractions (60% MVC). However, regional variations in MG architecture investigated at rest were not observed.

### Amplitude distribution of gastrocnemius activity during sub-maximal, isometric contractions

**Fig 3:**
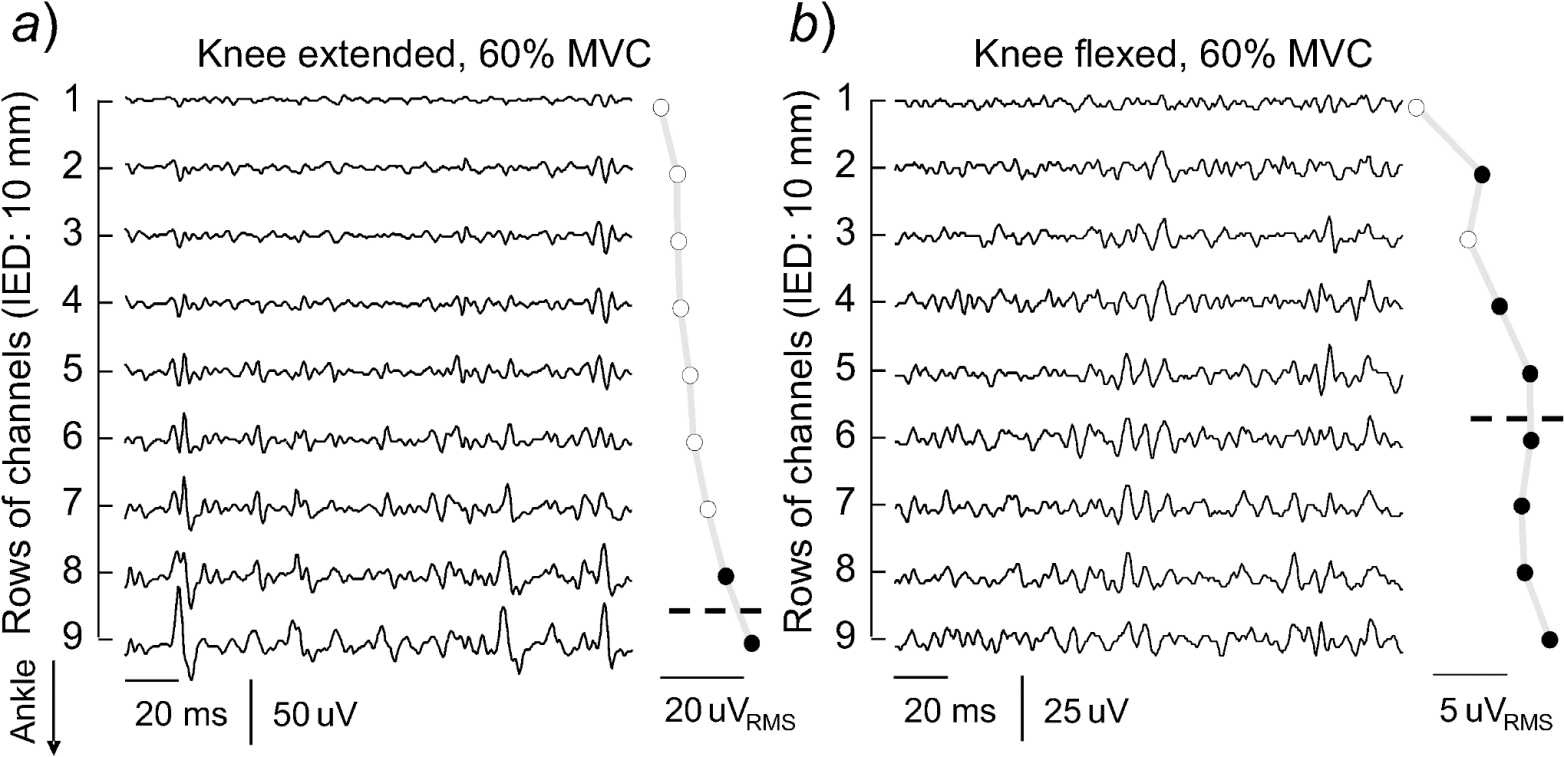
Changes in the surface EMGs with changes in knee position. A short epoch of raw, surface EMGs is shown during plantar flexion contractions exerted with the knee fully extended (*a*) and the knee flexed at 90 deg (*b*). Only nine of the 15 channels in the array were positioned on skin regions covering the MG superficial aponeurosis. The RMS amplitude computed from EMGs detected by each of these nine channels is shown on the right side of each panel, with black circles denoting the channels providing RMS amplitudes greater than 70% of the maximum. Dashed lines indicate the barycentre coordinate computed for these channels.

Surface EMGs detected along the MG muscle during knee extended and flexed positions were markedly different. As shown in Fig 3 for a representative participant, these differences manifested in the amplitude of surface EMGs and in its distribution. For the knee extended condition, relatively larger action potentials were observed in the more distal MG regions (cf. the amplitude of surface EMGs detected by different channels in Fig 3*A*). Consequently, greatest RMS values were obtained for the two most distal channels; these channels provided RMS values greater than 70% of the maximum RMS value in the grid (Fig 3*A*). For the knee flexed position, on the other hand, the RMS amplitude of surface EMGs distributed somewhat evenly across channels in the array; seven out of the nine channels located over the MG superficial aponeurosis for this subject provided similarly large RMS values (Fig 3*B*).

**Fig 4:**
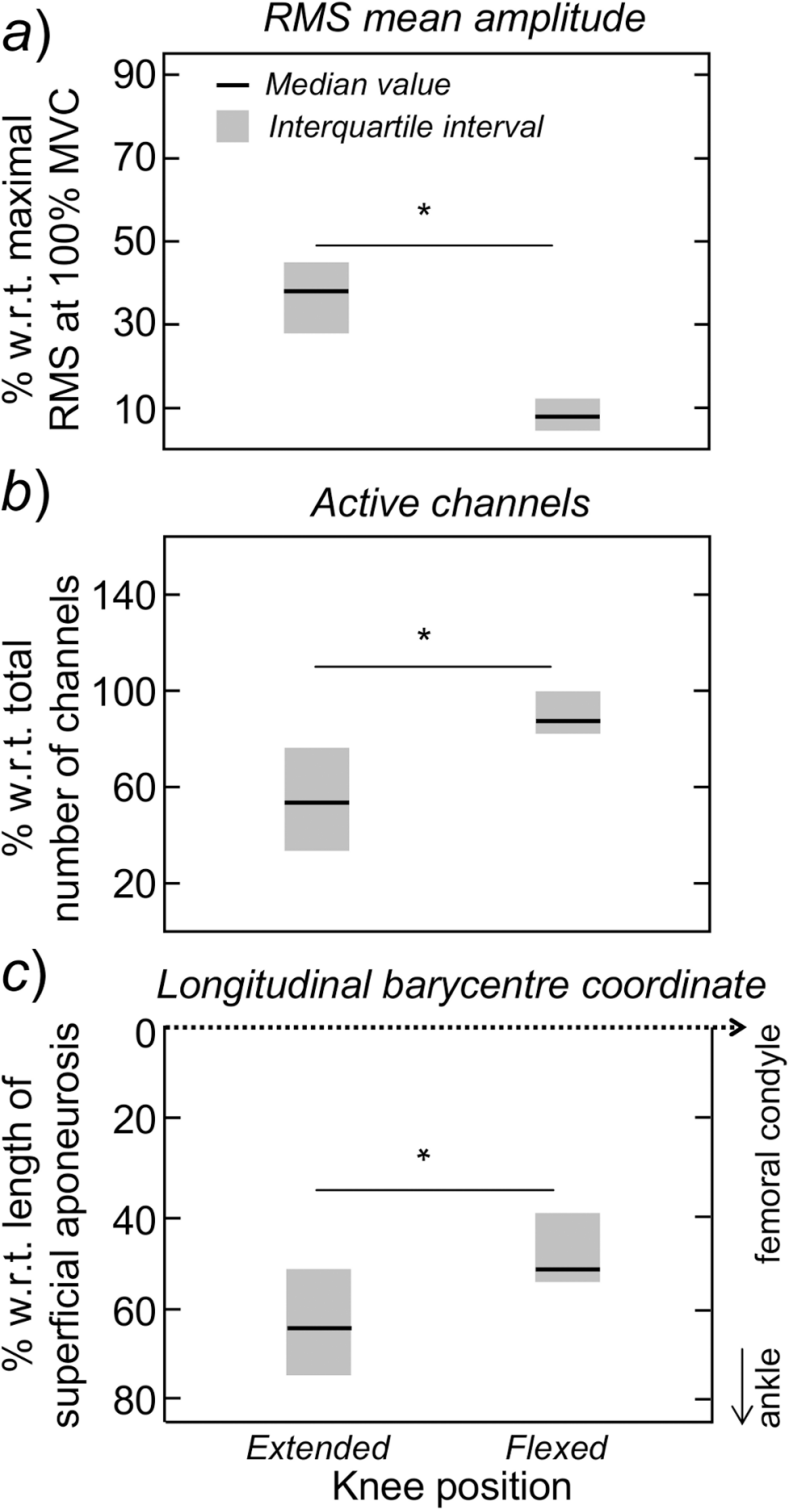
Changes in the spatial distribution of RMS values with knee position. Median values and inter-quartile intervals are shown for the RMS amplitude (*a*), the *active channels* (*b*) and the barycentre coordinate (*c*). These variables were respectively normalised with respect to the maximal RMS value obtained at 100% MVC attempts performed during knee extended position, the total number of channels located over the MG superficial aponeurosis and the distance between the femoral condyle and the distal extremity of the superficial aponeurosis (see Fig 1). Asterisks denote statistical significance at *P*<0.05.

The differences in EMG amplitude shown in Fig 3 were consistently observed across the 22 participants tested. The normalised, mean RMS amplitude observed during the knee extended position (inter-quartile interval: 28–45%) was approximately five times higher than that observed for the knee flexed position (4–12%; Fig 4*A*; Wilcoxon test; *P* = 0.001; *N* = 44; 22 subjects x 2 knee positions). The spatial distribution of RMS amplitude was however significantly more diffused on the skin during knee flexed than extended position. With knee extended, the relative number of *active channels* (33–75%) was significantly smaller than that obtained with knee flexed (81–100%; Fig 4*B*; Wilcoxon test; *P* = 0.001). Finally, the barycentre coordinate obtained for knee extended position was located at significantly more distal regions than that obtained for knee flexed position (Wilcoxon test; *P* = 0.001). For the knee flexed and extended conditions, the barycentre median position was located at respectively 50% (39–53%) and at 63% (50–74%) of the distance from the femoral condyle to the distal extremity of the MG superficial aponeurosis (Fig 4*C*).

### Gastrocnemius architectural changes revealed from ultrasound images at rest

**Fig 5:**
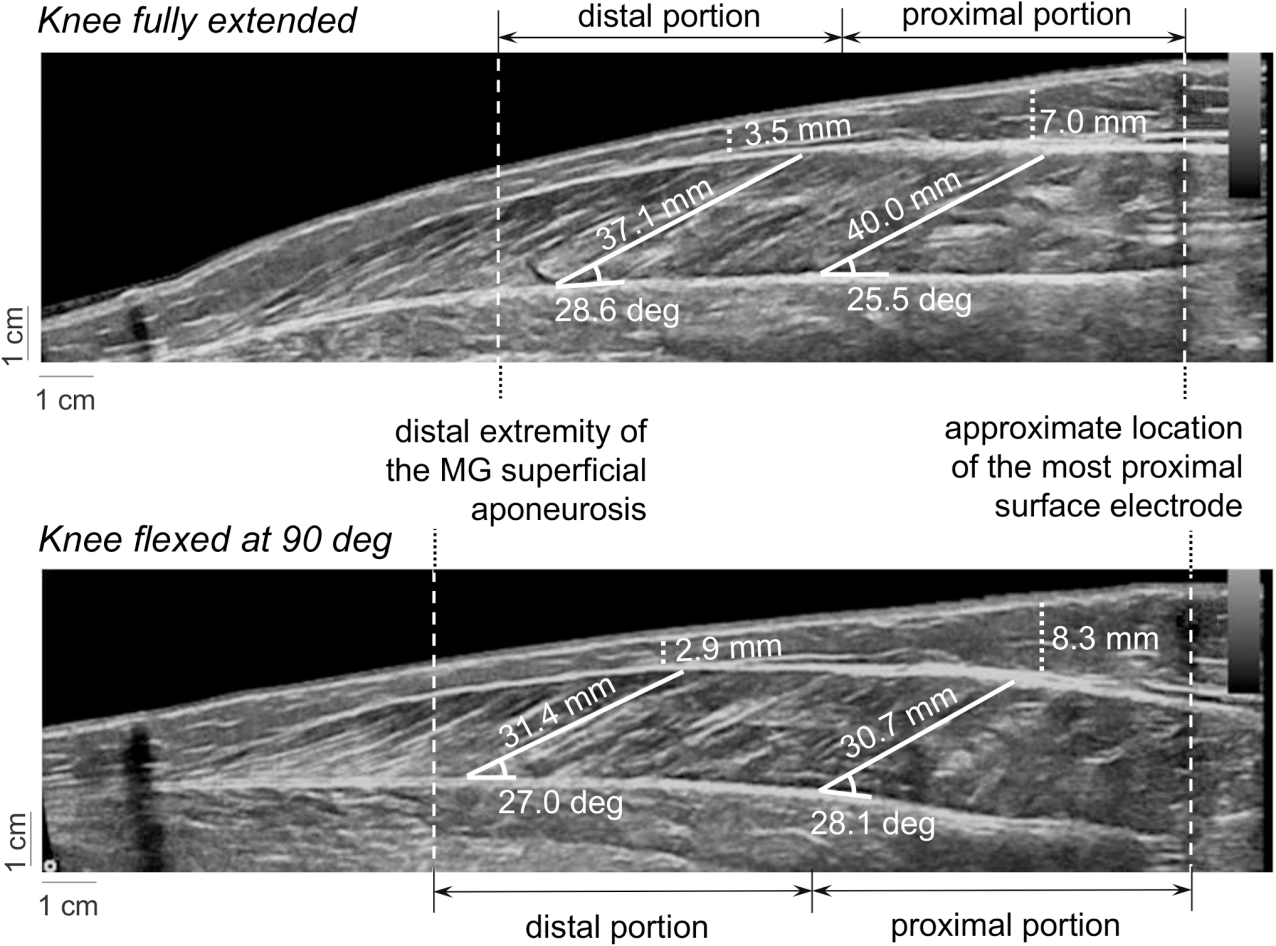
Ultrasound images and gastrocnemius architecture. The images shown in the top and bottom panels were collected with knee fully extended and flexed at 90 deg, respectively. Dashed lines superimposed on the images indicate the MG portion analysed, from the distal extremity of the superficial aponeurosis to the position of the most proximal electrode (see Fig 1). Dotted lines indicate estimates of fat thickness. Pennation angles were estimated from each pair of white, solid lines; these lines were placed in correspondence of the deep aponeurosis and MG fascicles.

Marked differences in MG architecture were observed when subjects moved their knee from extended to flexed position. As schematically illustrated in Fig 5 for a single, representative subject, the total muscle portion considered for analysis (Fig 1) was larger for knee flexed than extended condition. Specifically, the position of the distal extremity of the superficial aponeurosis shifted towards the most distal electrode with knee flexion (cf. the distance between the dashed, vertical lines shown in Fig 5). Moreover, flexing the knee from full extension to 90 deg led to a decrease in the length of MG fibres for both portions. Changes in knee position seem however to have affected more markedly the fibre length than the fat thickness and pennation angle. Although the fat thickness did not show significant changes with knee position, it was greater at the proximal than at the distal region (Fig 5).

**Fig 6:**
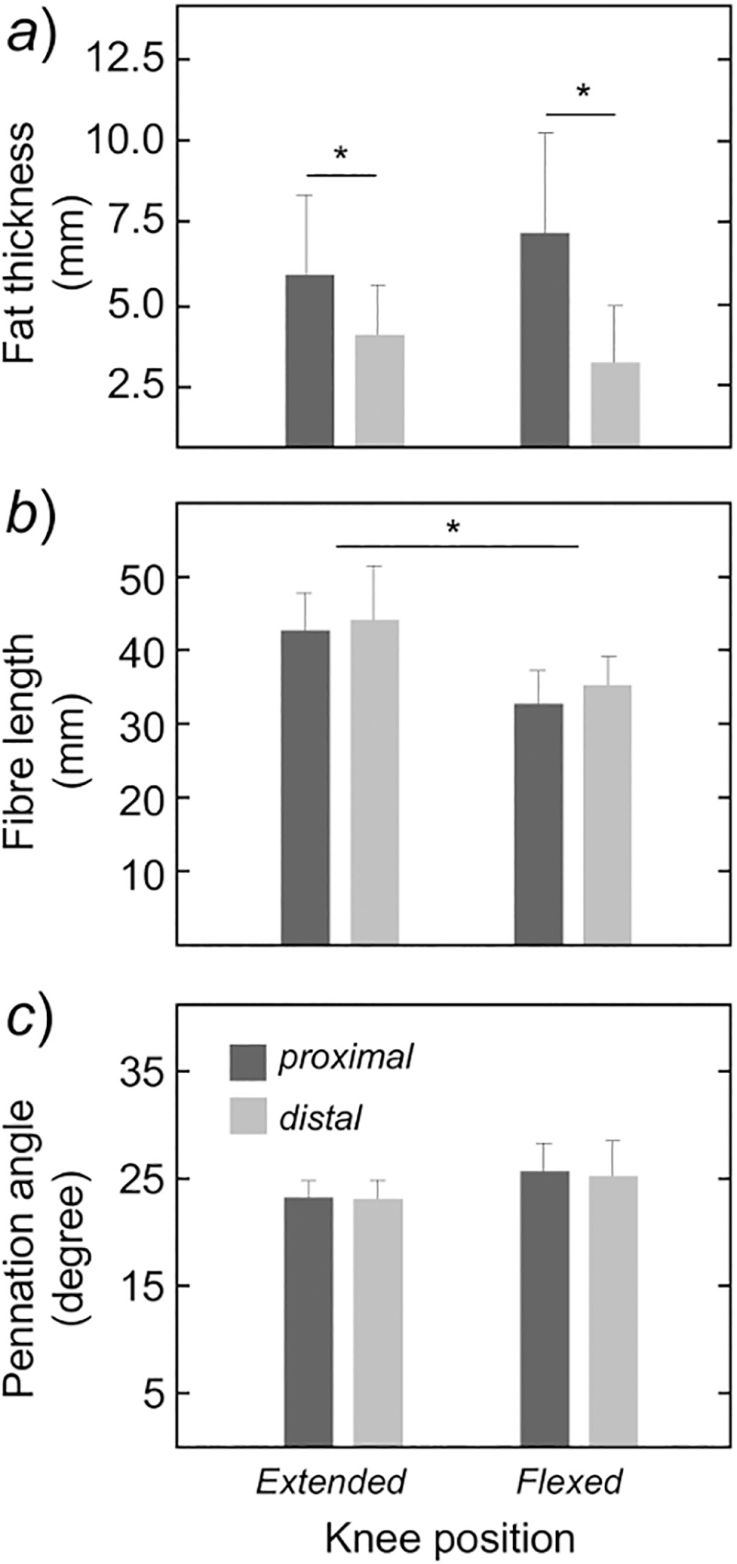
Regional changes in gastrocnemius architecture with knee position. Mean values and standard deviation (whiskers) are shown for the fat thickness (panel *a*), the MG fibre length (panel *b*), and their pennation angle (panel *c*). These values were obtained from panoramic ultrasound images (see Fig 5), separately for the proximal (dark, shaded bars) and distal (light, shaded bars) muscle regions. Asterisks denote statistical differences at *P* < 0.05.

When considering all participants, the proximo-distal differences in MG architecture were not significantly associated with knee position. With knee extended, the fat tissue was significantly thicker proximally (6.5 ± 2.4 mm) than distally (3.5 ± 1.9 mm; Fig 6*A*; Tukey HSD post-hoc, *P*<0.001). Similarly, for the knee flexed position, estimates of fat thickness (7.4 ± 3.4 mm; Fig 6*A*) obtained from the MG proximal region were significantly greater than those obtained from the distal region (3.3±2.3mm; Tukey HSD post-hoc; *P* < 0.001). Regardless of the MG region considered, the proximal (32.0 ± 5.6 mm) and distal (34.0 ± 5.4 mm) values obtained for MG fibre length with knee flexed were significantly smaller than those observed for the proximal (42.0 ± 6.8 mm) and distal (43.2 ± 8.5 mm) MG regions with knee extended (Fig 6*B*; ANOVA main effect, *P* < 0.001 for all cases). The proximal-distal differences in fat thickness and fibre length did not change however with knee position (ANOVA interaction effect; *P* > 0.37 *N* = 88; 22 subjects x 2 MG regions x 2 knee positions). No significant interaction or additive effect of knee position and/or muscle region was observed for the MG pennation angle (Fig 6*C*; ANOVA main and interaction effects; *P* > 0.27 for all cases).

## Discussion

In this study we investigated whether changes in the amplitude distribution of surface EMGs detected from the MG muscle were associated with knee position. We further assessed whether variations in EMG amplitude may be explained by MG architectural changes. Our main finds revealed that: i) the distribution of EMG amplitude along the skin surface during isometric, sub-maximal contractions changed markedly with knee flexion; ii) proximo-distal differences in MG fibre length and pennation angle, as well as in the fat thickness, whenever present, were not affected by knee position with the muscle at rest. Presuming the lack of architectural differences observed at rest within the muscle extends to the 60% MVC isometric contractions, these results suggest the redistribution of activity within the MG muscle, resulting from knee flexion, is unlikely related to anatomical factors.

### EMG amplitude distribution rather than EMG amplitude is affected by knee position

When flexing the knee by 90 deg from full extension, the amplitude of surface EMGs decreased markedly. Even though our subjects sustained plantar flexion torque at the same, relative effort level (60% MVC), the RMS amplitude of surface EMGs detected from the MG muscle was significantly lower with knee flexed than extended (Fig 4*A*). This observation is in agreement with previous accounts reporting diminished EMG amplitude in the MG muscle during isometric contractions performed with knee flexed [3, 11, 25]. A common explanation for this reduction in EMG amplitude is the distribution of the neural input to ankle plantar flexors according to their mechanical efficiency [3, 4]. With knee flexion, the gastrocnemius fibres shorten from their optimal length whereas the length of soleus fibres changes marginally [4, 26]. It is therefore reasonable to expect the gastrocnemius muscle to be activated to a lesser degree with knee flexion than other plantar flexors. As we recorded EMGs exclusively from the MG muscle, we could not verify whether the decrease in RMS amplitude observed for MG was compensated by increased EMG amplitude in e.g., soleus muscle. It must be noted however we were focused on the distribution of activity within the MG muscle rather than on the load sharing between plantar flexor synergists. From our results, indeed, it seems questionable whether descriptors of EMG amplitude (e.g., RMS, average rectified value, and others) sufficiently characterise the changes in the neural drive to plantar flexors with knee flexion.

In addition to changes in the degree of MG activity, knee flexion seems to lead to a redistribution of activity within the MG muscle. With knee extended, surface EMGs with greater RMS amplitude were detected by a few channels, located at the more distal MG regions. During knee flexed, notwithstanding their smaller RMS amplitude in relation to knee extended position, surface EMGs with relatively greater RMS amplitude were observed over a larger, and more proximal, skin region (Fig 4*C*–4*B* *)*. These differences in the amplitude distribution of surface EMGs must be interpreted with respect to the MG pinnate architecture. From skin parallel-fibred muscles, the spread of the RMS amplitude distribution of surface EMGs reflects the length and the orientation of muscle fibres [27]; in this case, surface EMGs detected by an array of electrodes sample from different, longitudinal sections of the same muscle fibres. From muscles pinnate in depth direction, the distribution of RMS amplitude on the skin surface indicates the location and the number of active fibres within the muscle [19, 28]; in this case, surface EMGs detected by electrodes positioned consecutively over the muscle superficial aponeurosis sample from different muscle fibres. Presumably, therefore, results presented in Fig 4 suggest a marked difference in the distribution of active fibres within the MG muscle for different knee joint angles. With the knee fully extended, isometric plantar flexions seem to demand activation of fibres grouped at the MG distal region (Fig 3*A*). At the 90 deg knee flexed position, the active fibres seem to spread within the MG muscle, spanning a large proximo-distal region (Fig 3*B*). With different methodologies, other researchers obtained direct evidence on the uneven distribution of active fibres within the MG muscle, both during isometric [18] and dynamic plantar flexions [13]. A corollary of current and previous findings is that the degree of MG activity, and by degree we refer to the relative amount of active MG fibres, cannot be inferred exclusively from a given RMS amplitude; the relative number of MG active fibres is not directly related to the amplitude of surface EMGs detected on a small skin region. While this remains the subject of future investigations, here we are concerned with the potential causes and implications of the redistribution of MG activity with knee position.

### Architectural differences within the gastrocnemius muscle unlikely explain the changes in activation with knee position

Previous researchers reported an uneven variation of MG fibre length in dynamic contractions. Lichtwark and collaborators, for instance, observed greater fascicle shortening at the more distal MG regions during walking [12]. Calf raising exercises seem to also demand a greater shortening-lengthening of the more distal MG fascicles [29]. It is therefore possible that the distribution of activation within the MG muscle and, thus, the distribution of EMG amplitude across channels in the array, could be shaped by proximo-distal differences in fibre shortening resulting from knee flexion. Potentially, in view of the MG force-length curve, the MG regions showing greater reductions in EMG amplitude with knee flexion would correspond to those exhibiting greater fibre shortening. Results shown in Figs 4 and 6, however, do not support this possibility. The amplitude of surface EMGs detected at the more distal MG regions decreased more strongly with knee flexion (Fig 4). If such uneven reduction in EMG amplitude was associated with fibre length, we would expect the fascicles residing in the MG distal region to shorten to a greater extent than the MG proximal fascicles when knee position changed from full extension to 90 deg flexion. Conversely though, and in agreement with Shin and colleagues [30], with knee flexion, fascicles at the MG proximal and distal regions shortened by statistically equal amounts (Fig 6*B*). These results do not exclude a possible relationship between whole-MG fibre shortening and regional changes in MG activation, as discussed in the next subsection. Results presented in this study, on the other hand, do not support the hypothesis that regional changes in MG fibre length account for the regional changes in MG activation with knee flexion.

Alternative hypotheses positing the effect of anatomical factors on surface EMGs also do not explain the uneven variations in EMG amplitude observed from knee extended to flexed position. In the literature, it is well established that changes in EMG features may be not exclusively related to alterations in the neural input to pools of motor neurons of a given muscle [21, 31]. The thickness of fat tissue and the pennation angle, for example, may affect markedly the amplitude of surface EMGs. Theoretical and experimental accounts have, indeed, shown the amplitude of surface EMGs decrease with the thickness of the fat tissue interposed between the target muscle and the skin [21, 32, 33]. For the 22 subjects tested in this study, the fat tissue covering the MG muscle was thicker at the more proximal regions (Fig 6*A*). On one hand, this suggests the amplitude of surface EMGs detected proximally was more attenuated by the fat tissue than that of EMGs recorded distally; i.e., the distance between electrodes and the superficial aponeurosis covering the proximal fascicles is greater proximally than distally. On the other hand, the proximo-distal difference in fat thickness did not depend on the knee position (Fig 6*A*). More specifically, the proximo-distal degree of attenuation of EMG amplitude, associated with the regional differences in fat thickness, unlikely explains the proximo-distal changes in RMS amplitude with knee position (Figs 3 and 4). A similar reasoning applies to MG pennation angle. Sadly, the effect of pennation angle on the amplitude distribution of surface EMGs is not well documented as the fat tissue effect is. Preliminary empirical data seems though to confirm theoretical evidence suggesting the spread of EMG amplitude distribution on the skin decreases with increases in pennation angle, presuming a constant, neural drive to the MG muscle [19]. In any case, regardless of the knee position considered, we did not observe significant proximo-distal differences in pennation angle within the MG (Fig 6*C*) when the muscle was at rest. Moreover, and contrarily to previous reports on MG architectural changes during concentric contractions [11], differences in pennation angle from knee extended to flexed position, with MG at rest, did not reach statistical significance. Divergences between studies could be possibly related to methodological issues; in our study, an extended field of view was provided by the panoramic, ultrasound images and architectural measurements were made at rest. Collectively, rather than spurious changes in the amplitude distribution of surface EMGs, our findings indicate that changes in knee position leads to a genuine alteration of the distribution of activity within the MG muscle during 60% MVC, isometric contractions.

### What is the origin for the redistribution of activity within the gastrocnemius muscle with knee flexion?

Different mechanisms could have contributed to triggering variations in activity within the MG muscle as the knee joint changed from extended to flexed position. The muscle mechanical efficiency, which is directly related with the length of MG fibres, has been suggested a crucial mechanism accounting for reduced MG activation with knee flexion [9, 26]. If fibre length at rest was the key mechanism underpinning changes in MG activation with knee position, then, the spatial distribution of RMS values across the muscle (Fig 3) should not change; i.e., general decrease of fibre length within MG (Figs 5 and 6) should lead to a general rather than localised decrease in RMS amplitude. According to our results, indeed, the contribution of fibre length to shaping MG activation seems less relevant than previously suggested. These results are in agreement though with the findings reported by Arampatzis and colleagues [25]. By mobilising the knee and ankle joints, Arampatzis et al. [25] observed significant reductions in the amplitude of surface EMGs collected from the gastrocnemius muscle without a corresponding, significant change in MG fibre length. It is therefore possible that sources other than fibre length substantially contribute to determining the net activation of the bi-articular, MG muscle. A potential candidate for sensing variations in knee joint and then providing key feedback information for the redistribution of activity within MG are the Achilles tendon receptors. During knee flexion, as shown in Fig 5 and as shown by others, the MG myotendinous junction moves distally. Such distal shift progressively unloads the Achilles tendon, possibly explaining the increased muscle-tendon compliance with knee flexion [34]. Considering the Achilles tendon compliance amounts to ~72% of the total muscle-tendon compliance [35], stiffening the Achilles tendon may thus be as important as, or perhaps more important than, relying on the MG fibre length for shaping whole muscle activation with knee flexion. In this view, distributing activity within the whole MG proximo-distal axis rather than within the MG most distal region (Figs 3 and 4) possibly optimises whole MG shortening and then Achilles tendon stiffening. In agreement with this hypothesis, with respect to rest condition, other researchers have reported greater increases in Achilles tendon length and greater shortening of the whole MG muscle when plantar flexion contractions were exerted with the knee in more flexed positions [36]. In this study we did not evaluate variations in Achilles tendon length during rest and during contractions. However, our results suggest that, although the force-length curve may explain the reduced ankle torque with knee flexion [3], it unlikely exclusively accounts for the changes in activity within the MG muscle during isometric contractions performed at different knee positions. Here we anticipate that in addition to MG fibres length, the degree of tendon slackness may potentially constitute a crucial source of feedback for the distribution of activity within the MG muscle.

### Limitations

Three limitations must be considered when interpreting our main results. First, ultrasound images were taken during rest and not during the isometric contractions. If MG proximal and distal fibres shortened by different amounts from rest to 60% MVC, then, the regional variations in EMG amplitude reported here may, at least in part, have been associated with regional changes in fibre length. On the other hand, given that proximo-distal differences in fibre length were not observed between knee flexed and extended position at rest (Figs 5 and 6), and considering the lack of evidence on regional variations in MG fibre length from rest to contraction [4, 10, 25], it is possible that any regional variation in MG fibre length occurring from rest to 60% MVC has manifested equally for both knee positions. Moreover, even if MG fibre length and activity had been measured concurrently, advancing cause-and-effect relationships between regional variations in architecture and activity would be still speculative. This issue is nevertheless being investigated with a recent technique developed in our laboratory for the simultaneous collection of US images and EMGs from the same muscle portion [37]. Second, fibre length estimation from conventional US images has many limitations, especially when the fibre is longer than the probe. As obtaining a reliable estimation of fibre length is of great importance to our study, we decided to collect panoramic rather than conventional US images. Panoramic images allow for the quantification of the actual fibre length without extrapolations [38, 39], at the cost of demanding greater expertise for image collection than the conventional procedure. In our study, US panoramic images were however collected by a well-trained experimenter. Finally, the results reported on the EMG amplitude distribution and MG architecture may not apply to conditions other than isometric contractions. Differences in the location where greatest EMGs were detected have been observed in a number of circumstances and for a number of muscles. Specifically for the gastrocnemius muscle, regional differences in EMG amplitude have been reported for different directions of ankle force [16], during quiet standing [14] and during fatiguing contractions [17, 18]. In the present study we were however focused on understanding whether knee joint position is an additional factor accounting for regional differences in EMG amplitude. Had we tested our subjects during walking or during a more functional situation, in view of these previous results, it would not be possible to separate the effect of knee position from other sources (e.g., force direction) on the EMG amplitude distribution.
